# Sequencing airborne DNA to monitor crop pathogens and pests

**DOI:** 10.1016/j.isci.2025.112912

**Published:** 2025-06-16

**Authors:** Amanda Mikko, Jose Antonio Villegas, Daniel Svensson, Edvin Karlsson, Per-Anders Esseen, Benedicte Riber Albrectsen, Ola Lundin, Mats Forsman, Anna Berlin, Per Stenberg

**Affiliations:** 1 Umeå Plant Science Centre, Department of Plant Physiology, Umeå University, Umeå, Sweden; 2 Department of Ecology and Environmental Sciences, Umeå University, Umeå, Sweden; 3 CBRN Defence and Security, Swedish Defence Research Agency (FOI), Umeå, Sweden; 4 Department of Ecology, Swedish University of Agricultural Sciences, Uppsala, Sweden; 5 Department of Forest Mycology and Plant Pathology, Swedish University of Agricultural Sciences, Uppsala, Sweden

**Keywords:** Environmental monitoring, Environmental biotechnology, Omics, Genomics, Agricultural science

## Abstract

Crop pests and diseases increasingly challenge the global food system. To prepare for and detect outbreaks, surveillance plays an important role. Traditional monitoring methods are often organism-specific, making large-scale monitoring of crop pathogens and pests impractical. We here investigate the potential for using shotgun sequencing of airborne eDNA for large-scale surveillance of crop pathogens and pests. We show that it is possible to detect DNA from all types of organisms in air, and that DNA can be classified down to species level. However, the accuracy of the identification is highly dependent on the quality of reference genomes of both the pathogens or pests, and their close relatives present in the region. Finally, we find that observed degree of crop damages correlate with amount of DNA from crop pathogens and pests in air, showing the promise of this approach for surveillance of all types of crop pathogens and pests.

## Highlights

• Airborne eDNA enables non-targeted monitoring of crop pathogens and pests
• Detection sensitivity and accuracy depend on quality of reference genome databases
• Airborne eDNA levels from pathogens and pests correlate with observed crop damage

## Graphical abstract

**Figure:**
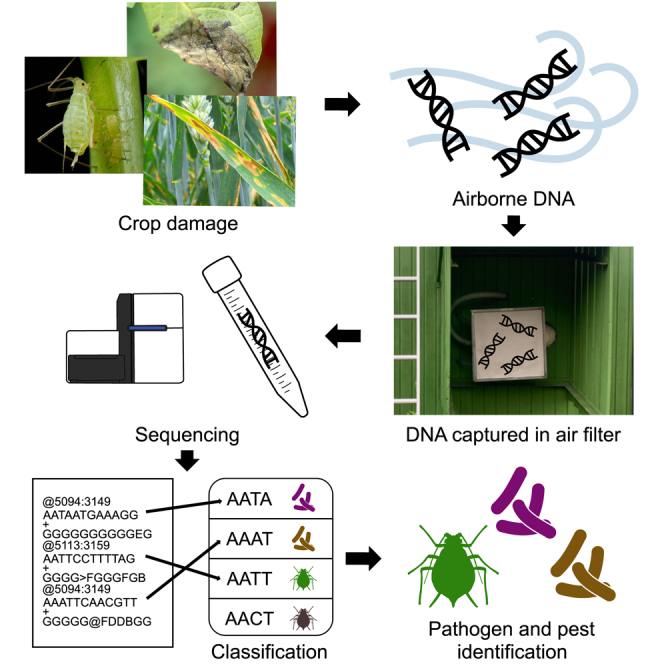

## Introduction

Plant diseases and pests reduce crop yields by 20%–30% globally, despite protection efforts.^1^^,^^2^ Monitoring plays an important role for understanding and predicting dispersal of pests and pathogens.^3^ For example, early detection of crop pathogens and pests is crucial to reduce the risk for outbreaks and allow for proactive prevention based on predictive models and forecasts.^4^

Monitoring aims to detect, diagnose, and quantify pests and diseases and is usually carried out by personnel who identify pests and diseases during field surveys or receive samples brought to a laboratory.^5^ To support diagnosis based on symptoms or organism characterization, molecular diagnostics has revolutionized the identification of pathogens and pests in recent decades.^6^ Furthermore, recent technological innovations allow us to monitor pathogens and pests through remote sensing (smart phone) image analysis,^7^^,^^8^^,^^9^^,^^10^ field sensors detecting volatile compounds,^11^^,^^12^ and a range of nucleic acid-based detection methods, including DNA sequencing technologies.^6^^,^^13^^,^^14^^,^^15^ For example, DNA sampled from the environment (eDNA) has been shown to have potential for monitoring of plant pathogens and pests as reviewed in Kestel et al.^16^

Using eDNA for monitoring purposes has been shown to have several advantages over traditional monitoring methods. For example, eDNA can speed up identification of insects^17^ and viruses,^18^ be used for early detection of invasive species,^19^ and used to identify crop pathogens on secondary plant hosts where they might otherwise have been overlooked.^20^

Previous studies investigating applications for eDNA in agriculture have mainly focused on DNA collected from soil and plant substrates.^16^ However, airborne DNA is a source of eDNA that has high potential for being used for monitoring purposes. For pathogen detection, airborne DNA has mainly been used to detect fungi and bacteria.^21^^,^^22^^,^^23^^,^^24^^,^^25^^,^^26^ But it has also been shown that air contains DNA from a wide range of other organisms such as insects, mammals, amphibians, and other vertebrates.^27^^,^^28^^,^^29^^,^^30^^,^^31^^,^^32^^,^^33^ Furthermore, it has been demonstrated that DNA abundance in air correlate well with observed abundances of birds.^32^ This displays the potential for using airborne eDNA for surveillance of wide ranges of organisms affecting crop health.

Most previous studies using airborne DNA for monitoring of crop pathogens and pests have used metabarcoding.^22^^,^^25^^,^^26^^,^^33^^,^^34^ A limitation with metabarcoding is that it targets specific taxa, meaning that protocols need to be adapted depending on the organism group. Thus, monitoring the wide range of pathogens and pests affecting crops quickly become unfeasible. If shotgun sequencing of eDNA can be used for surveillance purposes, it would allow for surveillance of all types of pathogens and pests. Indeed, it has been shown that it is possible to identify a number of pathogens and pests when shotgun sequencing DNA sampled from relatively small amounts of air (∼10 m^3^) in a crop field.^35^ Here we shotgun sequence DNA from air filters that filters approximately four orders of magnitude more air, and thus allow for detection of DNA originating from larger source areas.^32^ This is done to further investigate the potential for using shotgun sequencing of airborne DNA for monitoring of all types of crop pathogens and pests at a regional scale. We here show that DNA from a wide range of taxonomic groups containing pathogens and pests can be detected using shotgun-sequenced DNA from air filters collected in an agricultural area in southern Sweden. Furthermore, we investigate the conditions necessary for accurate species level classification and show that the observed eDNA abundance correlate to observed degrees of abundance or damage of pests and diseases in crops in the area.

## Results

### Air sampling

**Figure 1:**
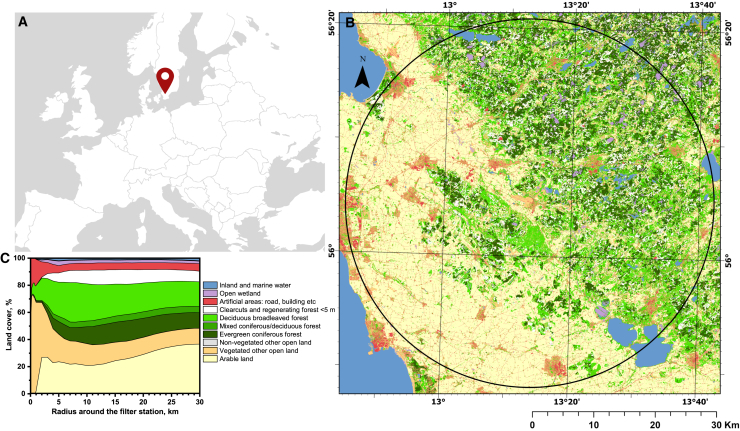
Geographical position of the air filter station and land cover within a 30 km radius (A) Map of Europe showing the location of Ljungbyhed in southernmost Sweden where eDNA was collected. (B) Map of ten land cover classes within a 30 km radius from the air filter station. (C) Percent land cover within circular buffers from 0.1 to 30 km distance around the air filter station.

We used air filters from a continuously operating filter station (Figure S1) located in Ljungbyhed, Sweden (lat 56.08°, long 13.23°).^36^ The filters are part of a larger collection that the Swedish Defense Research Agency (FOI) has gathered weekly since 1974 to monitor radioisotopes. Ljungbyhed is in the temperate (nemoral) zone in the county Skåne (Figure 1A). The land cover within 30 km radius from the air filter (Figure 1B) is dominated by arable land (36.8%), followed by deciduous forest (18.2%; with *Fagus sylvatica* dominant), vegetated other open land (11.7%; mainly grazing land), and coniferous forest (11.4%; mainly planted *Picea abies*). The landcover within 1 km from the air filter is dominated by vegetated other open land (65.4%) and artificial areas (19.2%; Figure 1C). Arable land is scarce within 1 km from the air filter (2.0%) but increases steeply up to 2 km (26.9%). The proportion of arable land decreases to 20.9% at ∼10 km distance and then gradually increases up to 30 km, whereas forests decrease.

Air filters were changed weekly, and they filter more than 100,000 m^3^ of air each. In total, nine air filters from 2007 were sequenced in this study. In a previous study, we have shown that these filters preserve DNA well over at least 50 years.^32^ The filters were collected during the cropping season when major crop pathogens and pests are expected to be present in the region. From each air filter, between 182 and 294 million paired-end reads (average: 212 million reads) were obtained. The number of sequenced reads from each week can be found in Data S1.

### A wide variety of pathogens and pests can be detected using airborne eDNA

To know what pathogens and pests could be of interest to detect in the air filters, we first compiled a list of current and potential European pathogens and pests by combining existing lists of pathogens and pests from the European and Mediterranean Plant Protection Organization (EPPO),^37^^,^^38^^,^^39^ the Swedish Board of Agriculture,^40^ the Northern Tubers of Potato Network,^41^ and Berlin et al.^42^ The compiled list consisted of 264 species with available reference genomes (in GenBank, NCBI, retrieved August 2022).^43^ The full list of species can be found in Data S1.

**Figure 2:**
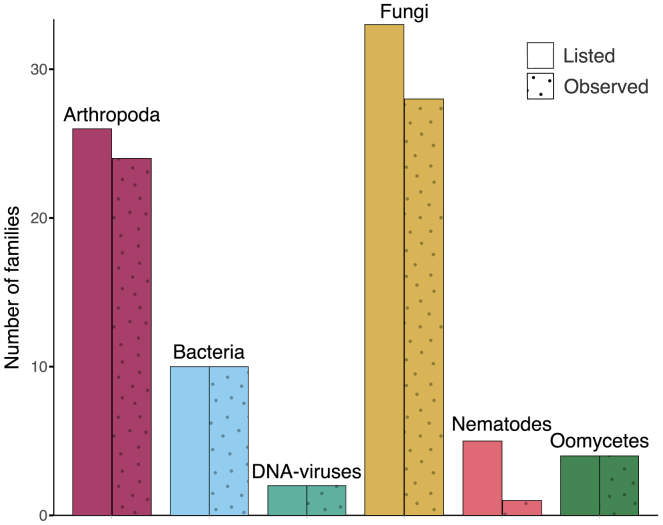
Number of detected taxonomic families containing pathogens or pests Detected taxonomic families containing pathogens or pests. Colors distinguish taxonomic groups. For each taxonomic group, the left bar represents the number of families in the taxonomic group that contain a species on our list of European pathogens and pests. The right bar represents the number of those families that were detected in the air filters. A family is considered detected if at least 1,000 reads is classified to the family from sequenced reads pooled from all air filters.

An initial analysis of what types of crop pathogens and pests could be detected in the eDNA dataset was performed using Kraken2^44^ with a custom reference database (see STAR Methods for details). From the combined classification results of all sequenced air filters, we detected a signal from most families known to contain pathogens or pests (Figure 2). This shows that many organism groups containing pathogens or pests shed DNA into the environment, and that this DNA can be detected in air. Notably, there is a lower proportion of nematode families detected, compared to other types of organisms. This could be due to a number of reasons (see below).

In the county where the air filters were collected, the Swedish Board of Agriculture reported presence or crop damage in 2007 from 13 species of pathogens and pests with a sequenced genome.^45^ Consequently, we analyzed if DNA from the reported species could be detected in the air filter data. To reduce the number of false classifications, we used the stringency parameters described in Sullivan et al.^32^ After pooling all sequenced reads from all air filters, we detected a signal from 11 out of the 13 species, and more than 1,000 reads from 6 of these species (Table 1).

**Table 1:** Summary of crop pathogens and pests observed by the Swedish Board of Agriculture in the county Skåne 2007 in relation to number of assigned DNA sequence reads

Species	Common name	Taxonomic group	Host	Total number of detections (no. field visits)	Average observation value	Number of reads	Fraction of total reads (1.9 × 10^9^)
*Pyrenophora teres*	net blotch	Fungi	Poaceae	313 (543)	18% affected leaves	174,190	9.3 × 10^−5^
*Blumeria graminis*	powdery mildew	Fungi	Poaceae	699 (1758)	24% affected leaves	145,829	7.7 × 10^−5^
*Zymoseptoria tritici*	septoria tritici blotch	Fungi	Poaceae	770 (872)	25% affected leaves	33,415	1.8 × 10^−5^
*Pyrenophora tritici-repentis*	tan spot	Fungi	Poaceae	82 (857)	7% affected leaves	12,671	6.7 × 10^−6^
*Acyrthosiphon pisum*	pea aphid	Arthropoda	Fabaceae	28 (56)	5 per top shoot	9,744	5.2 × 10^−6^
*Puccinia striiformis*	yellow rust	Fungi	Poaceae	121 (943)	15% affected leaves	2,833	1.5 × 10^−6^
*Brassicogethes aeneus*	common pollen beetle	Arthropoda	Brassicaceae	30 (49)	1 per plant	770	4.1 × 10^−7^
*Phytophthora infestans*	late blight	Oomycetes	Solanaceae	18 (54)	20% leaf surface	147	7.8 × 10^−8^
*Puccinia coronata*	crown rust	Fungi	Poaceae	2 (137)	2% affected leaves	72	3.8 × 10^−8^
*Puccinia hordei*	barley brown rust	Fungi	Poaceae	219 (543)	26% affected leaves	12	6.4 × 10^−9^
*Rhopalosiphum padi*	bird cherry-oat aphid	Arthropoda	Poaceae	160 (1,287)	0,2 per straw	5	2.7 × 10^−9^
*Metopolophium dirhodum*	rose-grass aphid	Arthropoda	Poaceae	33 (1282)	0,2 per straw	0	0
*Sitobion avenae*	grain aphid	Arthropoda	Poaceae	257 (1286)	0,3 per straw	0	0

The species are sorted based on total number of reads classified to the species using Kraken2 and our custom database (reads from all sequenced weeks were pooled). Total number of detections refers to the total number of times a non-zero value was reported for the pathogen or pest, with the total number of times sampled for within parenthesis. Average observation value refers to the average nonzero field observation value.

The varying degree of signal strength for the different species is likely due to a variety of reasons. For example, all species were not observed in equal frequency and intensity (Table 1). Different types of organisms might also shed different amounts of DNA into the environment and this DNA may vary in stability and dispersal in air. Database limitations is, however, likely the main factor causing weak signals for all species with less than 100 assigned reads (5 species).

If reads are generated *in silico* from the reference genomes of these five species with less than 100 reads, less than 1% of the generated reads are correctly classified using Kraken2 and our custom database (Figures S2–S14). This is potentially due to contaminated genomes and poor assemblies being included in the database. For example, reads generated from the reference genome of *Puccinia coronata* were misclassified to the mite *Medioppia subpectinata*. When these misclassified reads were mapped to the reference genome of *M. subpectinata*, we found that many regions where the generated reads mapped share high similarity with bacteria and fungi (using MegaBLAST^46^). This suggests that the genome for *M. subpectinata* is contaminated. Such contamination strongly interferes with Kraken2’s ability to classify reads since the software is dependent on correct taxonomic assignation of the reference DNA sequences.

### Detection sensitivity depends on database quality

Using Kraken2 to classify DNA from the air filters, we noted that seven species observed in crop fields had less than 1,000 detected reads in total. If these species were not detected due to database limitations, DNA from them could still be present in the air filters. To evaluate this possibility, we mapped all sequenced air filter reads to the individual reference genomes of each species with weak signal and evaluated the evenness of coverage. If DNA from a species is present in the air filters, we expect the DNA to have originated approximately randomly from across its genome. Consequently, we expect the mapped reads to be evenly distributed across the genome. This further means that the number of reads mapped to each scaffold should be strongly correlated to the scaffold length. To determine how strong this correlation must be for it to be likely that DNA from the species is present in the air filters, we compared it to the correlations produced by mapping *in silico*-generated reads from closely related species (same genera) to the same genome of interest. Doing this, we found that DNA from all species except *Sitobion avenae* likely is present in the air filters, since the air filter reads produced stronger correlations than the reads generated from close relatives (Figure S15). This shows that DNA from many of the species with weak signal likely is present in the air, but it is not possible to correctly classify the DNA using a general classifier such as Kraken2 and the current database.

### High quality databases enable accurate identification of pathogens and pests

For sequencing of air filters to be useful for monitoring purposes, it is not only important to detect signal from crop pathogens and pests. The detected signal also needs to be accurate. We therefore investigated the accuracy of the taxonomic classifications for the six pathogens and pests with more than 1,000 assigned reads (see Table 1). To do this, we evaluated how the classified reads for each species were distributed over what we define as classifiable regions of the genome. Classifiable regions are the unique regions of a genome that are not shared with other species in the reference database and, thus, from which it is possible to classify reads using Kraken2. If the classified DNA fragments originate from the correct species, we expected reads to be evenly distributed over the classifiable regions. We should thus see a strong correlation between the number of mapped reads per classifiable region and the length of the classifiable region.

**Figure 3:**
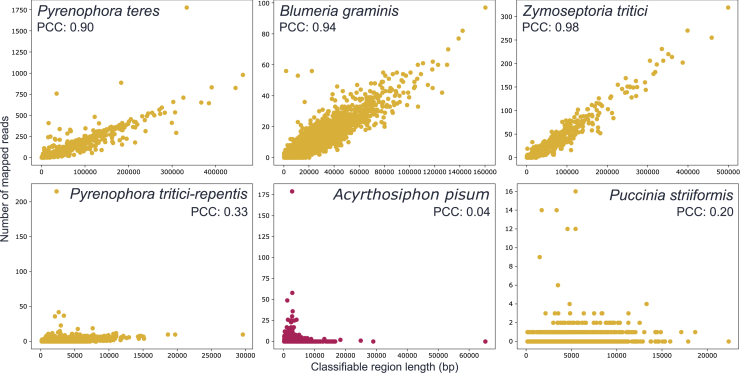
Classification quality vary depending on species Classifiable region length plotted against the number of air filter reads mapping to the region for all observed species with more than 1,000 assigned reads in DNA pooled from all sequenced air filters. Yellow species are fungi and red are arthropods. Classifiable regions were obtained by generating reads *in silico* from the species reference genome, classifying the generated reads, and then mapping the correctly classified reads back to the reference genome. Classifiable regions were then defined as regions of the reference genome with any coverage. The mapped reads consist of reads classified to the species using Kraken2 and our custom database; these reads were then mapped to the species reference genome. Strong correlations (PCC, Pearson Correlation Coefficient) indicate that most air filter reads were correctly classified, since the number of reads that aligns matches the expected number based on the length of the region, i.e., the reads are evenly distributed across the genome. Weak correlations point to lower classification accuracy since an uneven distribution across the genome indicate that some reads originate from, for example, mis-classifications or conserved regions of a closely related species that is missing from the database.

Using this approach, we noted that classification quality varies depending on species. *Pyrenophora tritici-repentis*, *Acyrthosiphon pisum*, and *Puccinia striiformis* likely have poor classification quality since we observed poor correlation values (Figure 3). This indicates that the classified reads contain misclassifications, possibly from related but not yet sequenced species containing highly similar genomic regions. Alternatively, these issues could be caused by contaminated genomes, low quality genomes, or DNA with incorrect taxonomic assignment being included in the used Kraken2 database. When reads were generated from the species of crop pathogens and pests with weak signal, it was noted that reads from other *Puccinia* spp. were misclassified as *Puccinia striiformis.* Reads from other aphids were also misclassified as *Acyrthosiphon pisum* (Figures S2–S14). These examples again illustrate the need of a highly accurate and curated database to avoid these misclassification issues.

For *Pyrenophora teres*, *Blumeria graminis*, and *Zymoseptoria tritici*, there is a strong correlation between the number of reads mapped to the classifiable regions and the length of the regions, showing that a large part of the reads is likely correctly classified. This shows that this type of classification can be done accurately for species for which previously mentioned database limitations can be avoided.

### Peak observation values coincide with increased air filter signal

**Figure 4:**
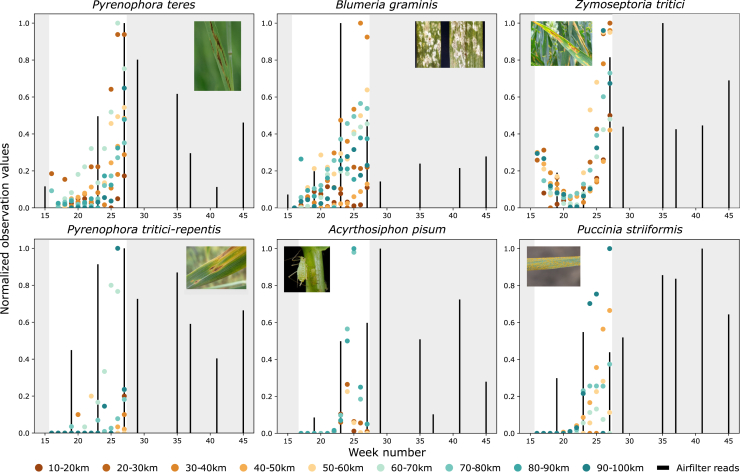
Observed abundance or damage caused by crop pathogens and pests correlate with air filter signal strength Plot of average observed abundance or observed damage caused by crop pathogens and pests (observation values) at different distance from the location of the air filter sampling station (colored by distance), and air filter signal strength (black bars). Crop pathogen and pest observation values from 2007 were obtained from the Swedish Board of Agriculture, and the reported unit varies depending on species (the same unit as the species average observation value in Table 1). Air filter signal strength for each species is represented by a logarithm ratio (pivot coordinate) to account for the inherent compositionality of the data. Both observation values and pivot coordinates were min-max normalized prior to visualization to make the largest value for each species equal to one. Gray areas show periods of time when no observation attempts were done. See also Figure S16.

By plotting average degree of observed abundance or damage caused by crop pathogens and pests together with detected signal strength, we found that the measurements correlate. For all the species with high confidence classifications, peak observation values coincide with increased signal strength (Figure 4). Furthermore, correlation between the values can also be seen for species with lower classification quality.

For *B. graminis*, air filter signal increases before peak observation values, indicating that air filters could be used to detect the pathogen before disease symptoms are observed in crop fields. For *A*. *pisum*, increased signal strength in the air filters is observed after peak observation values, indicating that aphid signal is strongest when crop damage have occurred and winged aphids leave the crop.^47^

This analysis further show that it is possible to use airborne DNA to monitor crop pathogens and pests, since air filter signal coincide with observation values or agree with the biology of the species. However, sequencing of additional weeks, preferably across several seasons, is necessary to evaluate the true correlation between observed abundance or damage by pathogens and pests to crops and the levels of their DNA in air.

## Discussion

We have shown that DNA from crop pathogens and pests is present in air and that the DNA obtained using non-targeted shotgun sequencing can be classified to species level, which was also recently shown by Giolai et al.^35^ The accuracy of the identification is highly dependent on the quality of the reference genomes of both the target species and their close relatives in the area. Poorly assembled and contaminated genomes severely impact our ability to link DNA signal to the correct species. However, detected eDNA signal strength is consistent with the biology of the pathogens and pests and correlates with the observed level of abundance or damage reported from crop fields in the area at the corresponding time of air sampling.

Of the six highly abundant pathogens and pests in air filters that were observed in crop fields in the area, five are pathogens causing economically important diseases in cereals, while the aphid pest *A*. *pisum* primarily causes damage in peas. All highly abundant pathogens are primarily wind-dispersed.^48^ For example, powdery mildew caused by *B. graminis* is mainly spread by wind-borne spores in early spring from winter wheat or volunteer plants to wheat crops. The disease develops during the growing season, when there is a peak in the air filter signal (Figure 3). After harvest, the pathogen survives on wheat volunteers and is spread by wind to new fields when autumn-sown wheat emerges.^48^ The pathogens causing leaf blotch disease in spring barley (*P. teres*) and wheat (*P. tritici-repens* and *Z. tritici*) are economically important and often sprayed with fungicides in the Nordic-Baltic countries.^49^ While prognosis models based on precipitation or humidity are available for leaf blotch diseases in cereals, these are not fully reliable.^50^^,^^51^ The cereal rust diseases, including yellow rust caused by *P. striiformis*, may cause severe yield losses in epidemic years. The rust diseases are transmitted by airborne spores and can disperse over long distances.^52^ Consequently, the sources of inoculum causing epidemics may be situated far from infected fields.^53^ As pest or disease occurrence in fields regionally matched with signal strength for pathogens and pests in the air filter, analysis of airborne eDNA represents a novel opportunity for surveillance of pests and diseases. Interestingly, eDNA abundance and the degree of damage or abundance in the crop fields seem to correlate even for the pathogens and pests where the classification quality was lower, possibly because the misclassified reads reflect closely related pests or pathogens with similar biology and phenology.

Using shotgun sequencing for surveillance allow for untargeted detection of a wide range of pests. Using this technique on air filters with high airflow enabling large catchment areas,^32^ could therefore be useful for large-scale surveillance of invasive species and emerging threats, such as quarantine plant pests. However, shotgun sequencing has certain disadvantages such as being more expensive and potentially less sensitive than PCR-based detection methods. After detection of threats, it could thus be more suitable to use local cheaper monitoring methods. For example, PCR-based monitoring methods that utilize air filters from filter stations with smaller throughput of air. Such a setup could take advantage of the strengths of both untargeted shotgun sequencing and targeted PCR-based methods.

The air filters examined here are from an archive of weekly filters, going back to the 1960s. If long time series are produced by sequencing more of these filters, information on emergence, seasonal patterns, and trends for crop pathogens and pests could be obtained, similar to those obtained for biodiversity monitoring in Sullivan et al.^32^ Furthermore, there are also other archives that could be sequenced, meaning that historic information on presence and dispersal of, for example, invasive species could be obtained from further sequencing efforts.^32^^,^^54^ Such data could be used for training of predictive models,^55^ which could be valuable tools used to strengthen food security. However, further research is needed to determine the catchment area when sampling airborne eDNA, since it will likely depend on, for example, the type of organism, weather conditions, the height of collection above the ground, and the volume of air sampled.

Taken together, we believe that it is valuable to continue research and development of DNA sequencing and subsequent classification of airborne DNA for surveillance and monitoring of crop pathogens and pests. Implementations of such technology would increase food security by improving our ability to monitor and predict abundance of crop pathogens and pests.

### Limitations of the study

The main limitation of this study is the limited number of samples (9 weeks from one year). Analysis of consecutive weeks, spanning multiple years would be necessary to further validate our observed correlation with observed crop damages. In addition, the available observational data on crop damages only partially overlap with our sampling period. A major limitation for the accuracy of assigning reads to the correct species is, as we here show, the quality of reference databases. With poor quality genomes and lack of reference genomes for closely related species present in the area, reads will be incorrectly classified. When more and better reference genomes become available, our data can easily be reclassified to improve accuracy and coverage of species.

## Resource availability

### Lead contact

Further information and requests for resources and reagents should be directed to and will be fulfilled by the lead contact, Per Stenberg (per.stenberg@umu.se).

### Materials availability

This study did not generate new unique reagents.

### Data and code availability

• Pre-processed shotgun sequence data from 9 air filters can be accessed from the Sequence Read Archive: PRJNA1173971.
• Code used for the analysis can be found in the Github repository: https://github.com/amandamikko/Sequencing-airborne-DNA-to-monitor-crop-pathogens-and-pests.
• Any additional information required to reanalyze the data reported in this paper is available from the lead contact upon request.

## Acknowledgments

This study was supported by 10.13039/501100001862Formas (grant agreement nos. 2016-01371 and 2019-00579) and Swedish Research Council (2021–06283). We would like to thank Alf Djurberg for providing observation data from the Swedish Board of Agriculture. We also acknowledge support from the 10.13039/501100009252Science for Life Laboratory and the National Genomics Infrastructure for providing assistance in massive parallel sequencing. The computations were enabled by resources provided by the National Academic Infrastructure for Supercomputing in Sweden and the Swedish National Infrastructure for Computing at UPPMAX and HPC2N partially funded by the 10.13039/501100004359Swedish Research Council (grant agreement nos. 2022–06725 and 2018–05973).

## Author contributions

P.S. and M.F. conceived and designed the study; E.K. extracted DNA; A.M. conducted most of the data analysis, with support from J.A.V., D.S., P.-A.E., B.R.A.; P.S. A.M. wrote the first draft of the manuscript, with support from O.L., A.B., and P.S. All authors contributed intellectual input and approved the final version.

## Declaration of interests

The authors declare no competing interests.

## STAR★Methods

### Key resources table

**Table:** 

REAGENT or RESOURCE	SOURCE	IDENTIFIER
**Biological samples**		

9 air filter samples. Ljungbyhed (Sweden, lat 56.08° long 13.23°)	This paper	N/A

**Chemicals, peptides, and recombinant proteins**		

MoBio PowerWater kit	MoBio Laboratories, Carlsbad, CA, USA	N/A
DNA Clean & Concentrator-5	Zymo Research, Irvine, USA	D4013

**Deposited data**		

Pre-processed shotgun sequence data from 9 air filters	This paper	Sequence Read Archive: PRJNA1173971

**Software and algorithms**		

ArcGIS Pro 3.3.0	Esri https://pro.arcgis.com/en/pro-app/3.3/tool-reference/main/arcgis-pro-tool-reference.htm	N/A
fastp 0.22.0	Chen et al.^56^ https://github.com/OpenGene/fastp	RRID:SCR_016962
BBMap 38.84	Bushnell et al.^57^ https://sourceforge.net/projects/bbmap/	RRID:SCR_016965
Kraken 2.1.2	Wood et al.^44^ https://github.com/DerrickWood/kraken2	RRID:SCR_026838
NEAT 3.3	Stephens et al.^58^ https://github.com/zstephens/neat-genreads	N/A
KronaTools 2.8	Ondov et al.^59^ https://github.com/marbl/Krona	N/A
Sankey 0.2	Breitwieser et al.^60^https://github.com/d3/d3-sankey	N/A
SAMtools 1.18	Li et al.^61^ https://github.com/samtools/samtools	RRID:SCR_002105
Picard 2.27.5	Picard Toolkit^62^ https://github.com/broadinstitute/picard	RRID:SCR_006525
zCompositions 1.4.0–1	Palarea-Albaladejo et al.^63^ https://cran.r-project.org/web/packages/zCompositions/index.html	N/A
R 4.2.1	R Core Team^64^ https://www.r-project.org	RRID:SCR_001905

**Other**		

Code used for the analysis	This paper. https://github.com/amandamikko/Sequencing-airborne-DNA-to-monitor-crop-pathogens-and-pests	N/A

### Method details

#### Land cover

Land cover data were extracted from the Swedish National Land Cover Database,^65^ mapped in 2017–2019 and with a pixel size of 10 m × 10 m. We extracted percent land cover in ten thematic classes within circular buffers of 0.1 km–30 km distance from the air filter station using Arc GIS Pro 3.3.0 (Esri). Forests outside and on wetlands were not separated.

#### DNA sequencing

Air filters were collected in Ljungbyhed (Sweden, lat 56.08° long 13.23°) in the county Skåne, southernmost Sweden. The filters are part of a larger collection that the Swedish Defense Research Agency (FOI) has gathered weekly since 1960’s to monitor radioisotopes (Figure S1). Filters are made of glass fiber with a pore size of 0.2 μm and filters air at a rate of 10L/min, meaning that more than 100,000 m^3^ of air is filtered through each filter (Camfil type CS 5.0, Camfil Svenska AB).^36^ In total nine air filters from 2007, processed in Karlsson et al.,^22^ were selected and sequenced. Filters were collected during the weeks described in the table below (Air Filter Collection, 2007, Ljungbyhed). The weeks were chosen to reflect key crop stages in the region. Weeks 15 and 19 correspond with the start of the growing season, when winter crops begin to grow and spring crops are sown. Weeks 23, 27 and 29 are during critical crop development stages; if a pest occurs at this time, direct control is required to ensure a high yield of good quality. Weeks 37 and 39 are when winter crops are sown and finally weeks 41 and 45 are in the autumn when winter crops have emerged and reflect pest pressure before winter dormancy.

DNA extraction was previously performed by Karlsson et al.^22^ using a modified MoBio PowerWater kit (MoBio Laboratories, Carlsbad, CA, USA) and blank filters as negative controls. In brief, three ∅8 mm punches (Integra Miltex, Plainsboro, NJ, USA) were taken from each filter and placed in 2mL tubes with a mix of zirconia/silica beads. After adding 1 mL preheated PW1 solution, samples were incubated at 65°C for 10min, agitated in FastPrep-24 (Mp Biomedicals, Santa Ana, CA, USA), and centrifuged. The supernatant was then transferred into a new tube. This process was repeated twice to yield three supernatants per filter piece. DNA was isolated following the manufacturers’ protocol, with pooled supernatants loaded onto a single spin filter. Finally, DNA from the three filter pieces were combined and concentrated into 75 μL elution buffer using a DNA Clean & Concentrsatior-5 kit (Zymo Research, Irvine, CA, USA). DNA samples were then sent for sequencing at the SNP&SEQ Technology Platform (SciLifeLab, Uppsala, Sweden) using the TruSeq Nano DNA library Prep kit with 100 ng input DNA and the HiSeq 2500 sequencing platform (150bp, paired-end) (Illumina, San Diego, CA, USA). Blank filter controls were not sequenced due to insufficient DNA concentration.

##### Air filter collection, 2007, Ljungbyhed

Information on when air filters were collected. Each row describes during what period each air filter was installed and filtering air 2007 in Ljungbyhed, Sweden (lat 56.08° long 13.23°).

**Table:** 

ISO week number	From	To
15	April 9	April 15
19	May 7	May 13
23	June 4	June 10
27	July 2	July 8
29	July 16	July 22
35	August 27	September 2
37	September 10	September 16
41	October 8	October 14
45	November 5	November 11

#### Read preprocessing and filtering

Adapters were removed from the sequenced reads, using fastp 0.22.0^56^ with the --detect_adapter_for_pe option. After removing adapters, all reads shorter than 50 base pairs were discarded. Furthermore, air filters were changed manually at the air filter stations. Therefore, we discarded all reads that mapped to the human reference genome hg19. Mapping was done using BBMap 38.84^57^ with the following parameters: minid: 0.95, maxindel: 3, minhits: 2, bandwidthratio: 0.16, bandwidth: 12, qtrim: “rl”, trimq: 10, quickmatch: “quickmatch”, fast: “fast”, untrim: “untrim”.

#### List of crop pathogens and pests

To obtain information about what organisms could be of interest to detect signal from in the air filters, a list of current and potential European pathogens and pests was compiled. This list was created from lists published by EPPO,^37^^,^^38^^,^^39^ lists from the Swedish Board of Agriculture,^40^ lists on potato pathogens and pests put together by experts associated with the NKJ network, Northern Tubers of potatoes (N’TOP),^41^ and a list of the most common pathogens and pests targeting field grown crops published by Berlin et al.^42^ The full list of pathogens and pests is presented in Data S1.

### Quantification and statistical analysis

#### Database creation and classification

Before subsequent analysis could be performed, the pre-processed reads needed to be classified to the organism they originate from. To do this a custom Kraken2^44^ database was created. Based on the created list of pathogens and pests, the database was created from sequenced species originating from the same taxonomic family as a sequenced crop pathogen or pest. This was done to distinguish DNA of pathogens and pests from that of other closely related species. All genomes available in GenBank were used to create the database for non-bacterial species. For bacteria, only the reference genomes were used for all species due to the large number of published genomes. This was done to keep the database smaller in size. All genomes used to create the database are found in Data S1. Furthermore, all DNA sequences included in the nt database provided by the National Library of Medicine^66^ 2022-12-20 was used to create the database. The nt database was used since it contains a wide variety of nucleotide sequences and can thus be used to increase the database’s general classification capability.

From all the mentioned DNA sequences, a database was built using Kraken 2.1.2 and the fast-build option. Minimizer and k-mer size settings were kept at their default values. This resulted in an 805 GB database. All reads were classified using the custom database with a confidence score of 0.1 and 10 minimum minimizer hit groups. These are the settings used by Sullivan et al.^32^ for genus level classification of reads sequenced from the same type of air filters sampled from Kiruna (Sweden, Lat.: 67.83650° Long.: 20.41582°). The parameter values were chosen since they provided the best trade-off between error rate and the number of classified reads.

#### Comparison to observation data

After classification, we investigated if a signal can be detected from pathogen and pest species that were observed in Skåne 2007. Information about what pests and diseases were observed in Skåne, were obtained from the Swedish Board of Agriculture.^45^ The reported data are based on weekly field inspections of untreated plots within commercial fields conducted by the staff of the Plant Protection Centers at the Swedish Board of Agriculture. The objective of these surveys is to provide farmers with information regarding the necessity of protecting their crops from pests and diseases using pesticides. Consequently, field surveys are conducted from crop emergence in May until the end of June, which corresponds with the main crop growth period and when pesticide treatments in crops are allowed.

#### Evaluation of classification potential

To evaluate our ability to classify reads from all observed species, we generated reads from the species reference genomes and attempted to classify the generated reads using our Kraken2 database. Used reference genomes is found in Data S1. For all species, 10x coverage was generated using NEAT (3.3)^58^ and the parameters: R: 126, c: 10, E: 0, pe, --force-coverage, average insert size 300, and standard deviation 30. Reads were then classified using our custom Kraken2 database with a confidence score of 0.1 and 10 minimum minimizer hit groups. Classification distribution were visualized using KronaTools (2.8)^59^ and Sankey (0.2).^60^

#### Mapping to determine if DNA from low-abundance species is present in the air filters

If DNA from a species is present in the air filters, we expect this DNA to originate approximately randomly from the genome. When mapping the air filter reads to the species genome, we would thus expect to see an even coverage across the genome. By evaluating coverage evenness, we should thus be able to determine if a species is likely present in the environment or not. However, coverage can be expected to be very low in our data, making it hard to evaluate directly. Due to this we instead evaluate the correlation between number of mapped reads per scaffold and scaffold length as a measurement for coverage evenness.

To determine if DNA from the low-abundance species is present in the air filters, we map all reads from all air filters to the reference genome of the species using BBMap (38.84) with the following parameters: minid: 0.97, ambigious: “toss”, pairedonly: “t”. We arbitrarily consider all species with less than a total of 1000 classified reads as low-abundant species. To avoid PCR duplicates and reads from genomic regions that are conserved between many species, we only consider reads that are overlapping with a maximum of one more read. From these reads, the Pearson correlation between number of mapped reads per scaffold and scaffold length was calculated. To determine how strong this correlation should be before DNA from a species can be regarded as present in the air filters, we generate reads from other sequenced species from the same taxonomic genus as the evaluated species. To generate these reads we use NEAT (3.3) with parameters: R: 126, c: 10, E: 0, pe, --force-coverage. Average insert size and standard deviation was estimated using SAMtools (1.18)^61^ on the air filter reads that mapped to the evaluated species. The generated reads were finally mapped using BBMap (38.84) and the same parameters used to map the sequenced air filter reads.

From the mapped generated reads, we then subsample the number of reads corresponding to the mapped air filter reads coverage of the evaluated species. Correlation between number of mapped reads per scaffold and scaffold length was then calculated and compared to the same correlation produced by the air filter reads. If the correlation produced by the air filter reads were stronger than the correlation produced by reads generated from all closely related species, DNA from the species was regarded as present in the air filters.

#### Evaluation of classification quality

To evaluate classification quality, we used a mapping approach where coverage evenness was evaluated. First, we mapped all reads from all air filters that were classified to a species to that species reference genome using BBMap (38.84) with the following parameters: minid: 0.97, ambiguous: “toss”, pairedonly: “t”. We then used Picard (2.27.5)^62^ to remove PCR duplicates from the mapped reads.

Not all regions of the genome can be classified using the Kraken2 database since only regions containing k-mers unique to the species can be used for classification. Because of this, we did not use coverage directly to evaluate how evenly reads map to the reference genomes. Instead, we first extracted the classifiable genomic regions containing species unique k-mers and then compared the length of these regions to the number of reads mapping to the region.

To find regions containing species unique k-mers, reads were first generated from the species reference genome. These reads were generated using NEAT (3.3) with parameters: R: 126, c: 10, E: 0, pe, and --force-coverage. Average insert size and standard deviation was approximated using SAMtools (1.18) on the mapped air filter reads that were classified to the species. The generated reads were then classified using Kraken (2.1.2) and our custom database, using the same parameters as when classifying air filter reads. To find the classifiable regions, the correctly classified reads were mapped to the reference genome from which they were generated. Mapping was done using BBMap (38.84) with the same parameters used to map the air filter reads. After mapping, the length of all regions with any coverage was extracted and considered to be classifiable genomic regions. The length of these regions was plotted against the number of air filter reads mapping to them, and the correlation between the two values was used to evaluate classification quality.

#### Correlation between observed abundance or damage and air filter abundance

Average reported abundance or damage for each species at different distances from the air filter station were plotted together with observed signal strength in the air filters to visualize how well the values correlated. Pivot coordinates (a type of logarithm ratio) were used to display air filter signal strength, to account for the inherent compositionality of the data.^67^ To calculate pivot coordinates, weekly number of air filter reads assigned to all species was used. Zero inflated taxa with more than ≥ 66.7% zero values were removed, zero replacement was then performed using geometric Bayesian multiplicative replacement^68^ as implemented in the cmultRepl function in the “zCompositions” R package.^63^^,^^64^ Pivot coordinates were finally obtained by calculating the first isomeric log ratio (ilr) coordinate for each $x=\left(x_{1},\ldots ,{\;x}_{D}\right)$ using the formula:

$$ilr\left(x\right)=z=\left(z_{1},...,z_{D-1}\right)$$

$$z_{j}=\sqrt{\frac{D-j}{D-j+1}}\ln\left(\frac{x_{j}}{\sqrt[{D-j}]{\prod_{k=j+1}^{D}x_{k}}}\right),for\;j=1,\ldots ,D-1$$

Where D is the number of parts in the composition.

Prior to visualization, all values were min-max normalized.

## Supplemental information

Document S1. Figures S1–S16

Data S1. Output from sequencing, list of pathogens and pests, list of reference genomes included in the database, and list of reference genomes used for evaluation of species presence and classification quality

## References

[1] Oerke E.C. (2006). Crop losses to pests. J. Agric. Sci..

[2] Savary S., Willocquet L., Pethybridge S.J., Esker P., McRoberts N., Nelson A. (2019). The global burden of pathogens and pests on major food crops. Nat. Ecol. Evol..

[3] Barzman M., Bàrberi P., Birch A.N.E., Boonekamp P., Dachbrodt-Saaydeh S., Graf B., Hommel B., Jensen J.E., Kiss J., Kudsk P. (2015). Eight principles of integrated pest management. Agron. Sustain. Dev..

[4] Parnell S., van den Bosch F., Gottwald T., Gilligan C.A. (2017). Surveillance to Inform Control of Emerging Plant Diseases: An Epidemiological Perspective. Annu. Rev. Phytopathol..

[5] Ristaino J.B., Anderson P.K., Bebber D.P., Brauman K.A., Cunniffe N.J., Fedoroff N.V., Finegold C., Garrett K.A., Gilligan C.A., Jones C.M. (2021). The persistent threat of emerging plant disease pandemics to global food security. Proc. Natl. Acad. Sci. USA.

[6] Silva G., Tomlinson J., Onkokesung N., Sommer S., Mrisho L., Legg J., Adams I.P., Gutierrez-Vazquez Y., Howard T.P., Laverick A. (2021). Plant pest surveillance: from satellites to molecules. Emerg. Top. Life Sci..

[7] Pethybridge S.J., Nelson S.C. (2015). Leaf Doctor: A New Portable Application for Quantifying Plant Disease Severity. Plant Dis..

[8] Ramcharan A., Baranowski K., McCloskey P., Ahmed B., Legg J., Hughes D.P. (2017). Deep Learning for Image-Based Cassava Disease Detection. Front. Plant Sci..

[9] Ramcharan A., McCloskey P., Baranowski K., Mbilinyi N., Mrisho L., Ndalahwa M., Legg J., Hughes D.P. (2019). A Mobile-Based Deep Learning Model for Cassava Disease Diagnosis. Front. Plant Sci..

[10] Mrisho L.M., Mbilinyi N.A., Ndalahwa M., Ramcharan A.M., Kehs A.K., McCloskey P.C., Murithi H., Hughes D.P., Legg J.P. (2020). Accuracy of a Smartphone-Based Object Detection Model, PlantVillage Nuru, in Identifying the Foliar Symptoms of the Viral Diseases of Cassava–CMD and CBSD. Front. Plant Sci..

[11] Li Z., Paul R., Ba Tis T., Saville A.C., Hansel J.C., Yu T., Ristaino J.B., Wei Q. (2019). Non-invasive plant disease diagnostics enabled by smartphone-based fingerprinting of leaf volatiles. Nat. Plants.

[12] Cui S., Ling P., Zhu H., Keener H.M. (2018). Plant Pest Detection Using an Artificial Nose System: A Review. Sensors.

[13] Mumford R., Boonham N., Tomlinson J., Barker I. (2006). Advances in molecular phytodiagnostics – new solutions for old problems. Eur. J. Plant Pathol..

[14] Adams I.P., Fox A., Boonham N., Massart S., De Jonghe K. (2018). The impact of high throughput sequencing on plant health diagnostics. Eur. J. Plant Pathol..

[15] Lau H.Y., Botella J.R. (2017). Advanced DNA-Based Point-of-Care Diagnostic Methods for Plant Diseases Detection. Front. Plant Sci..

[16] Kestel J.H., Field D.L., Bateman P.W., White N.E., Allentoft M.E., Hopkins A.J.M., Gibberd M., Nevill P. (2022). Applications of environmental DNA (eDNA) in agricultural systems: Current uses, limitations and future prospects. Sci. Total Environ..

[17] Kudoh A., Minamoto T., Yamamoto S. (2020). Detection of herbivory: eDNA detection from feeding marks on leaves. Environ. DNA.

[18] Boykin L.M., Sseruwagi P., Alicai T., Ateka E., Mohammed I.U., Stanton J.-A.L., Kayuki C., Mark D., Fute T., Erasto J. (2019). Tree Lab: Portable Genomics for Early Detection of Plant Viruses and Pests in Sub-Saharan Africa. Genes.

[19] Valentin R.E., Fonseca D.M., Nielsen A.L., Leskey T.C., Lockwood J.L. (2018). Early detection of invasive exotic insect infestations using eDNA from crop surfaces. Front. Ecol. Environ..

[20] Michael P.J., Jones D., White N., Hane J.K., Bunce M., Gibberd M. (2020). Crop-Zone Weed Mycobiomes of the South-Western Australian Grain Belt. Front. Microbiol..

[21] Banchi E., Ametrano C.G., Tordoni E., Stanković D., Ongaro S., Tretiach M., Pallavicini A., Muggia L., Tassan F., ARPA Working Group (2020). Environmental DNA assessment of airborne plant and fungal seasonal diversity. Sci. Total Environ..

[22] Karlsson E., Johansson A.-M., Ahlinder J., Lundkvist M.J., Singh N.J., Brodin T., Forsman M., Stenberg P. (2020). Airborne microbial biodiversity and seasonality in Northern and Southern Sweden. PeerJ.

[23] Métris K.L., Métris J. (2023). Aircraft surveys for air eDNA: probing biodiversity in the sky. PeerJ.

[24] Ovaskainen O., Abrego N., Furneaux B., Hardwick B., Somervuo P., Palorinne I., Andrew N.R., Babiy U.V., Bao T., Bazzano G. (2024). Global Spore Sampling Project: A global, standardized dataset of airborne fungal DNA. Sci. Data.

[25] Redondo M.A., Berlin A., Boberg J., Oliva J. (2020). Vegetation type determines spore deposition within a forest–agricultural mosaic landscape. FEMS Microbiol. Ecol..

[26] Tordoni E., Ametrano C.G., Banchi E., Ongaro S., Pallavicini A., Bacaro G., Muggia L. (2021). Integrated eDNA metabarcoding and morphological analyses assess spatio-temporal patterns of airborne fungal spores. Ecol. Indic..

[27] Polling M., Buij R., Laros I., de Groot G.A. (2024). Continuous daily sampling of airborne eDNA detects all vertebrate species identified by camera traps. Environ. DNA.

[28] Clare E.L., Economou C.K., Bennett F.J., Dyer C.E., Adams K., McRobie B., Drinkwater R., Littlefair J.E. (2022). Measuring biodiversity from DNA in the air. Curr. Biol..

[29] Garrett N.R., Watkins J., Simmons N.B., Fenton B., Maeda-Obregon A., Sanchez D.E., Froehlich E.M., Walker F.M., Littlefair J.E., Clare E.L. (2023). Airborne eDNA documents a diverse and ecologically complex tropical bat and other mammal community. Environ. DNA.

[30] Johnson M.D., Barnes M.A., Garrett N.R., Clare E.L. (2023). Answers blowing in the wind: Detection of birds, mammals, and amphibians with airborne environmental DNA in a natural environment over a yearlong survey. Environ. DNA.

[31] Lynggaard C., Bertelsen M.F., Jensen C.V., Johnson M.S., Frøslev T.G., Olsen M.T., Bohmann K. (2022). Airborne environmental DNA for terrestrial vertebrate community monitoring. Curr. Biol..

[32] Sullivan A.R., Karlsson E., Svensson D., Brindefalk B., Villegas J.A., Mikko A., Bellieny D., Siddique A.B., Johansson A.-M., Grahn H. (2023). Airborne eDNA captures three decades of ecosystem biodiversity. bioRxiv.

[33] Roger F., Ghanavi H.R., Danielsson N., Wahlberg N., Löndahl J., Pettersson L.B., Andersson G.K.S., Boke Olén N., Clough Y. (2022). Airborne environmental DNA metabarcoding for the monitoring of terrestrial insects—A proof of concept from the field. Environ. DNA.

[34] Nicolaisen M., West J.S., Sapkota R., Canning G.G.M., Schoen C., Justesen A.F. (2017). Fungal Communities Including Plant Pathogens in Near Surface Air Are Similar across Northwestern Europe. Front. Microbiol..

[35] Giolai M., Verweij W., Martin S., Pearson N., Nicholson P., Leggett R.M., Clark M.D. (2024). Measuring air metagenomic diversity in an agricultural ecosystem. Curr. Biol..

[36] Söderström C., S B., Jansson P., Lindh K., Tooloutalaie N. (2007). https://www.foi.se/en/foi/reports/report-summary.html?reportNo=FOI-R--2260--SE.

[37] European, Mediterranean Plant Protection Organization. EPPO A1 List of pests recommended for regulation as quarantine pests. https://www.eppo.int/ACTIVITIES/plant_quarantine/A1_list.

[38] European and Mediterranean Plant Protection Organization. EPPO A2 List of pests recommended for regulation as quarantine pests. (2022). https://www.eppo.int/ACTIVITIES/plant_quarantine/A2_list.

[39] European and Mediterranean (2022). Plant Protection Organization.EPPO Alert List.

[40] The Swedish Board of Agriculture (2021). Skadegörare i jordbruksgrödor.

[41] Northern Tubers of Potato https://nordicagriresearch.org/2020-13/.

[42] Berlin A., Källström H.N., Lindgren A., Olson Å. (2018). Scientific evidence for sustainable plant disease protection strategies for the main arable crops in Sweden. A systematic map protocol. Environ. Evid..

[43] Sayers E.W., Cavanaugh M., Clark K., Ostell J., Pruitt K.D., Karsch-Mizrachi I. (2020). GenBank. Nucleic Acids Res..

[44] Wood D.E., Lu J., Langmead B. (2019). Improved metagenomic analysis with Kraken 2. Genome Biol..

[45] Swedish Board of Agriculture (2023). Skadegörare på karta.

[46] Camacho C., Coulouris G., Avagyan V., Ma N., Papadopoulos J., Bealer K., Madden T.L. (2009). BLAST+: architecture and applications. BMC Bioinf..

[47] Brisson J.A., Stern D.L. (2006). The pea aphid, Acyrthosiphon pisum: an emerging genomic model system for ecological, developmental and evolutionary studies. Bioessays.

[48] Boland G., Agrios G.N. (2007).

[49] Jalli M., Kaseva J., Andersson B., Ficke A., Nistrup-Jørgensen L., Ronis A., Kaukoranta T., Ørum J.-E., Djurle A. (2020). Yield increases due to fungicide control of leaf blotch diseases in wheat and barley as a basis for IPM decision-making in the Nordic-Baltic region. Eur. J. Plant Pathol..

[50] Jørgensen L.N., Matzen N., Ficke A., Nielsen G.C., Jalli M., Ronis A., Andersson B., Djurle A. (2020). Validation of risk models for control of leaf blotch diseases in wheat in the Nordic and Baltic countries. Eur. J. Plant Pathol..

[51] Andersson B., Djurle A., Ørum J.E., Jalli M., Ronis A., Ficke A., Jørgensen L.N. (2022). Comparison of models for leaf blotch disease management in wheat based on historical yield and weather data in the Nordic-Baltic region. Agron. Sustain. Dev..

[52] Brown J.K.M., Hovmøller M.S. (2002). Aerial dispersal of pathogens on the global and continental scales and its impact on plant disease. Science.

[53] Lewis C.M., Morier-Gxoyiya C., Hubbard A., Nellist C.F., Bebber D.P., Saunders D.G.O. (2024). Resurgence of wheat stem rust infections in western Europe: causes and how to curtail them. New Phytol..

[54] Littlefair J.E., Allerton J.J., Brown A.S., Butterfield D.M., Robins C., Economou C.K., Garrett N.R., Clare E.L. (2023). Air-quality networks collect environmental DNA with the potential to measure biodiversity at continental scales. Curr. Biol..

[55] Emery S.E., Klapwijk M., Sigvald R., Bommarco R., Lundin O. (2023). Cold winters drive consistent and spatially synchronous 8-year population cycles of cabbage stem flea beetle. J. Anim. Ecol..

[56] Chen S., Zhou Y., Chen Y., Gu J. (2018). fastp: an ultra-fast all-in-one FASTQ preprocessor. Bioinformatics.

[57] Bushnell B. (2014). Conference: 9th Annual Genomics of Energy & Environment Meeting. Walnut Creek, CA, March 17-20, 2014.

[58] Stephens Z.D., Hudson M.E., Mainzer L.S., Taschuk M., Weber M.R., Iyer R.K. (2016). Simulating Next-Generation Sequencing Datasets from Empirical Mutation and Sequencing Models. PLoS One.

[59] Ondov B.D., Bergman N.H., Phillippy A.M. (2011). Interactive metagenomic visualization in a Web browser. BMC Bioinf..

[60] Breitwieser F.P., Salzberg S.L. (2020). Pavian: interactive analysis of metagenomics data for microbiome studies and pathogen identification. Bioinformatics.

[61] Li H., Handsaker B., Wysoker A., Fennell T., Ruan J., Homer N., Marth G., Abecasis G., Durbin R., 1000 Genome Project Data Processing Subgroup (2009). The Sequence Alignment/Map format and SAMtools. Bioinformatics.

[62] Picard Toolkit. (2019). https://broadinstitute.github.io/picard/.

[63] Palarea-Albaladejo J., Martín-Fernández J.A. (2015). zCompositions — R package for multivariate imputation of left-censored data under a compositional approach. Chemometr. Intell. Lab. Syst..

[64] R Core Team (2022).

[65] Naturvårdsverket. Nationella Marktäckedata (NMD). https://www.naturvardsverket.se/verktyg-och-tjanster/kartor-och-karttjanster/nationella-marktackedata.

[66] NCBI Resource Coordinators (2013). Database resources of the National Center for Biotechnology Information. Nucleic Acids Res..

[67] Filzmoser P., Hron K., Templ M. (2018).

[68] Martín-Fernández J.-A., Hron K., Templ M., Filzmoser P., Palarea-Albaladejo J. (2015). Bayesian-multiplicative treatment of count zeros in compositional data sets. Stat. Model. Int. J..

