# Supplementary material for: Sequencing airborne DNA to monitor crop pathogens and pests
Source: iScience. 2025 Jun 16;28(7):112912. doi: 10.1016/j.isci.2025.112912 (PMC12269458; doi:10.1016/j.isci.2025.112912)
Supplement: Document S1. Figures S1–S16 [file mmc1.pdf]

iScience, Volume *28*

# Supplemental information

# Sequencing airborne DNA to monitor crop pathogens and pests

**Amanda Mikko, Jose Antonio Villegas, Daniel Svensson, Edvin Karlsson, Per-Anders Esseen, Benedicte Riber Albrectsen, Ola Lundin, Mats Forsman, Anna Berlin, and Per Stenberg**

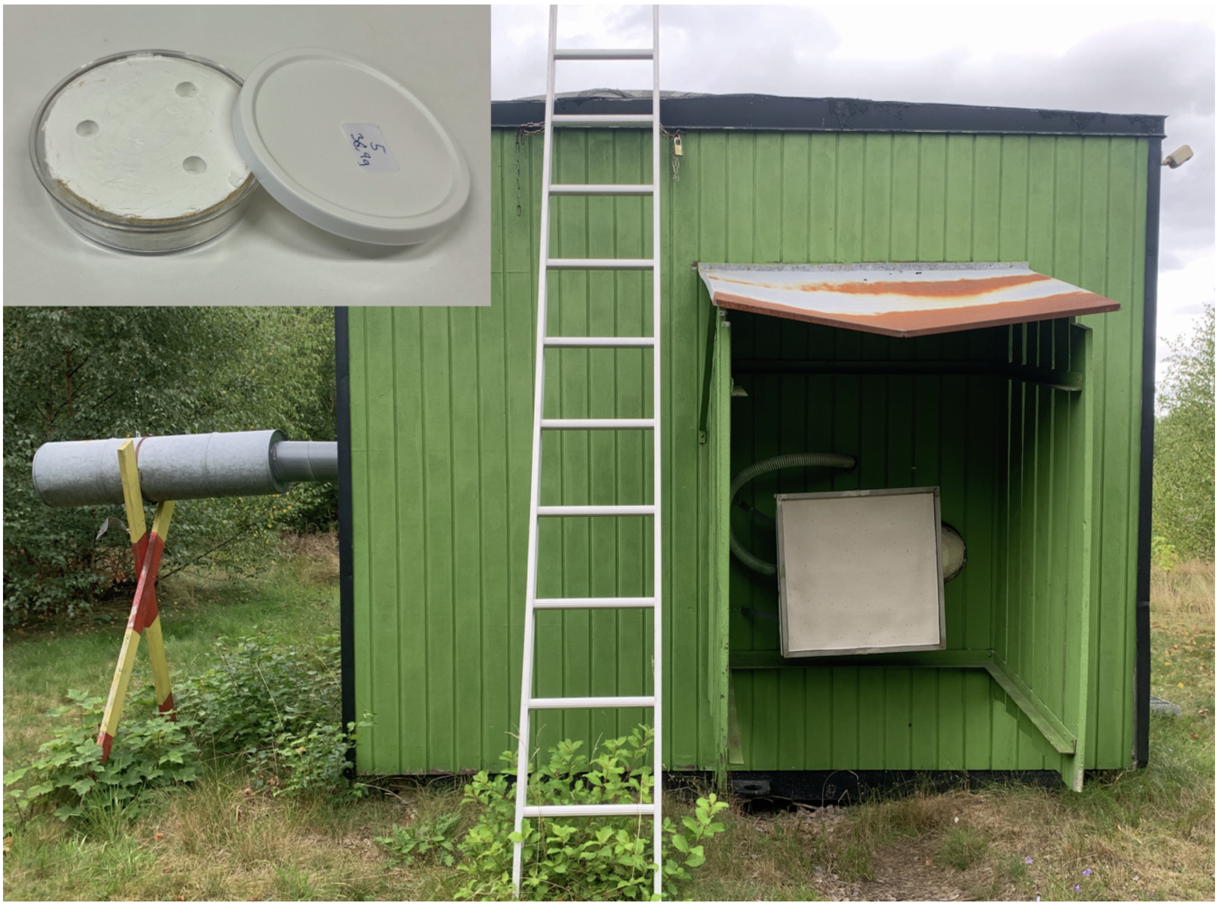

**Figure S1. Aerosol sampling station and compressed filter, Related to Figure 1.**
The 60 x 60 cm glass fibre-based filter is seen as a white square on the front side of the building. After a week mounted in the station, the filter is sent for analysis. Filters are first compressed to a cylinder, then analysed for radioactive isotopes, and finally archived in air-tight plastic containers (inset). The compressed filter in the picture is a control filter that has been processed the same way as the real samples. The three holes are from the three punches used for DNA extraction.

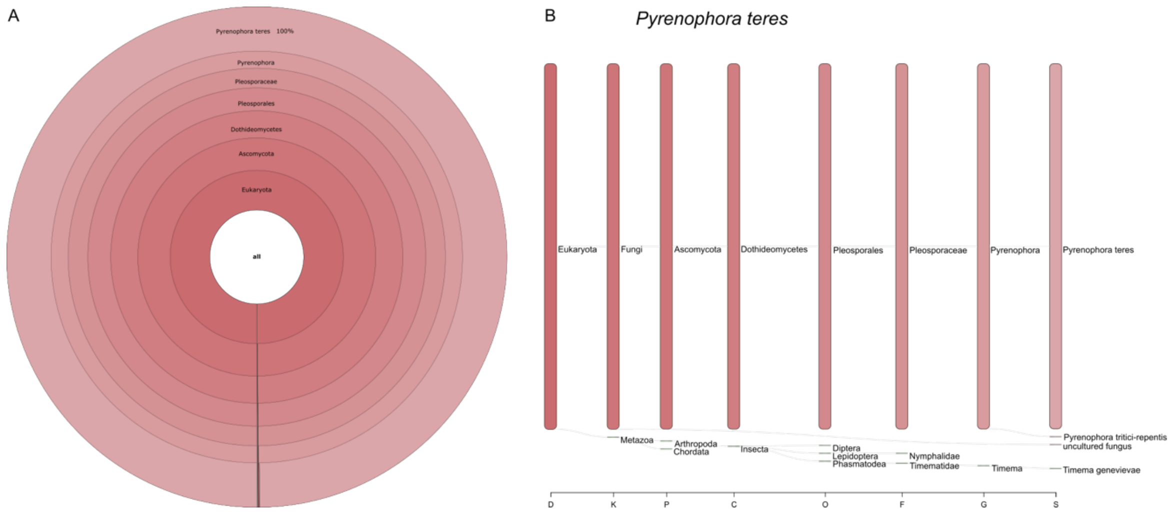


**Figure S2. Evaluation of our ability to classify DNA from *Pyrenophora teres*, Related to Table 1.**

Overview of how reads generated from the reference genome of *Pyrenophora teres* are classified using Kraken2 and our custom database. A) displays distribution of all classified reads B) displays how the classified reads are distributed.

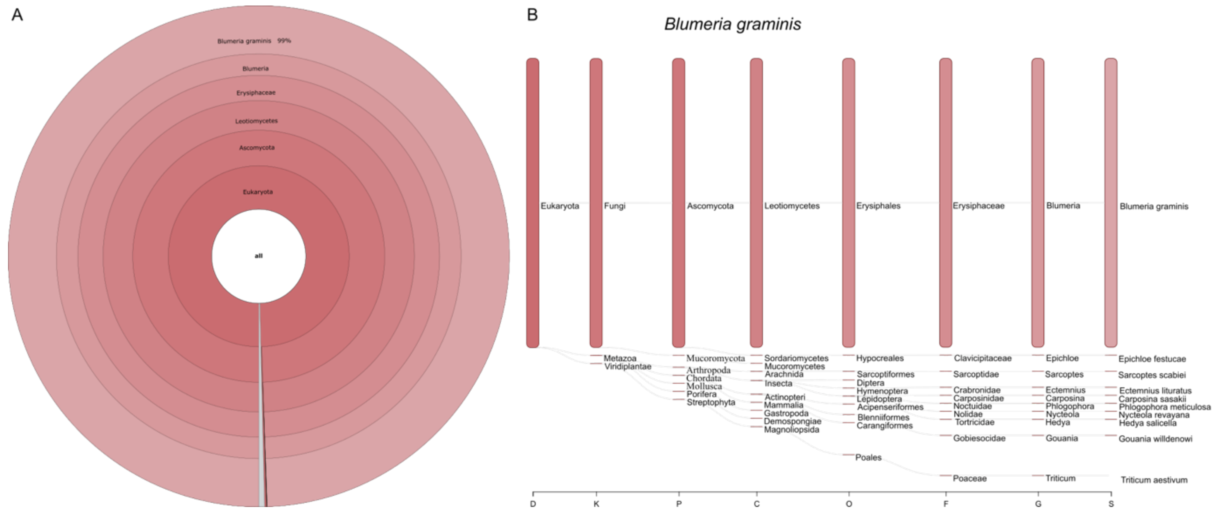


**Figure S3. Evaluation of our ability to classify DNA from *Blumeria graminis*, Related to Table 1.**

Overview of how reads generated from the reference genome of *Blumeria graminis* are classified using Kraken2 and our custom database. A) displays distribution of all classified reads B) displays how the classified reads are distributed.

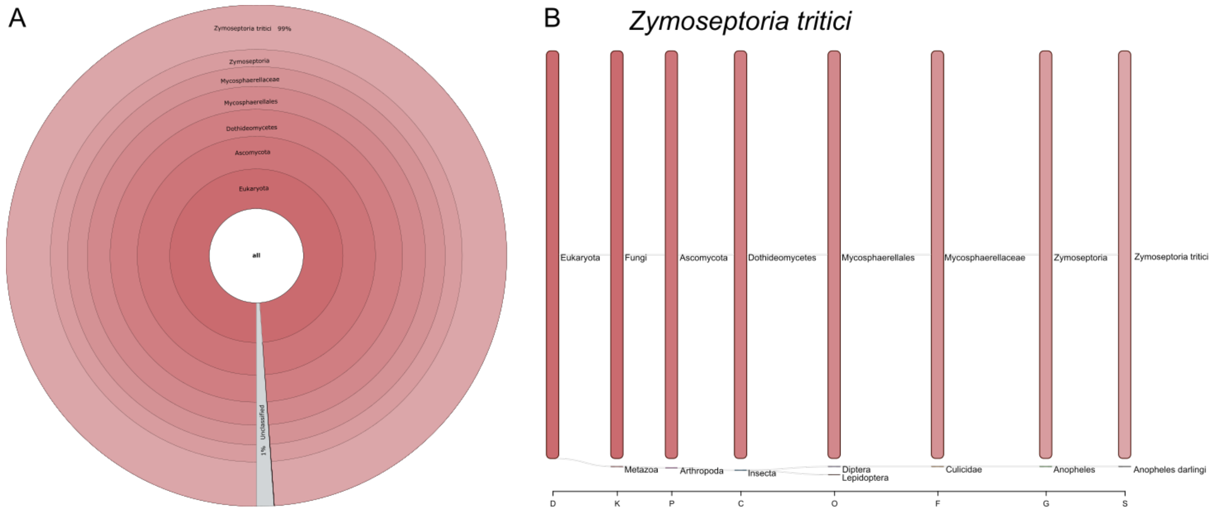


**Figure S4. Evaluation of our ability to classify DNA from *Zymoseptoria tritici*, Related to Table 1.**
Overview of how reads generated from the reference genome of *Zymoseptoria tritici* are classified using Kraken2 and our custom database. A) displays distribution of all classified reads B) displays how the classified reads are distributed.

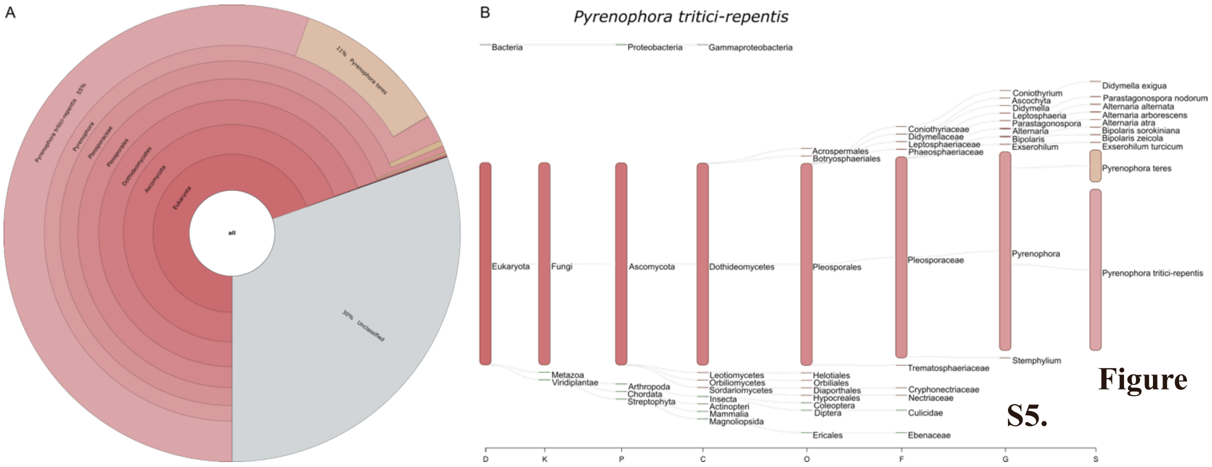


**Figure S5. Evaluation of our ability to classify DNA from *Pyrenophora tritici-repentis*, Related to Table 1.**
Overview of how reads generated from the reference genome of *Pyrenophora triticirepentis* are classified using Kraken2 and our custom database. A) displays distribution of all classified reads B) displays how the classified reads are distributed.

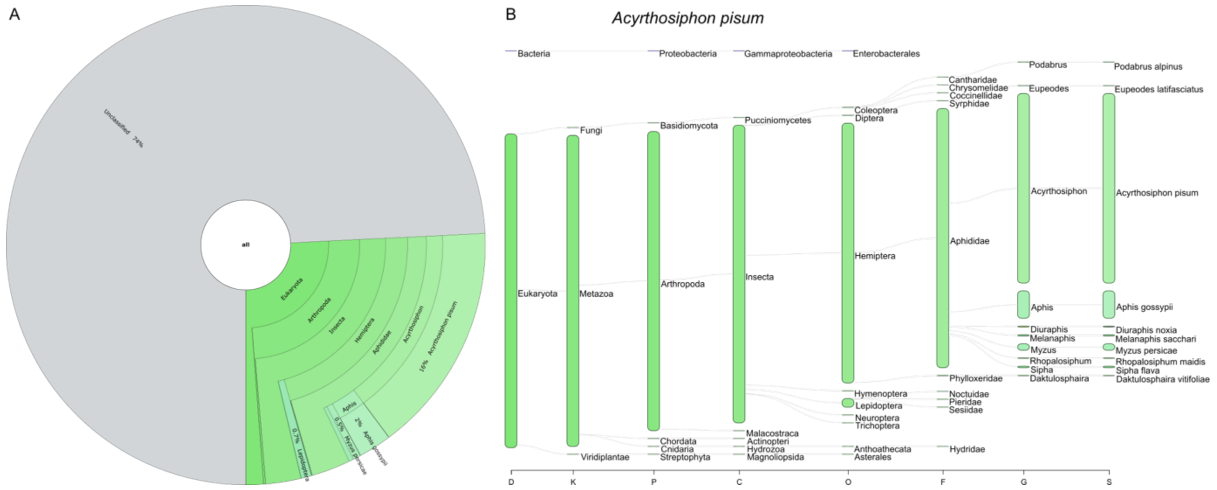


**Figure S6. Evaluation of our ability to classify DNA from *Acyrthosiphon pisum*, Related to Table 1.**

Overview of how reads generated from the reference genome of *Acyrthosiphon pisum* are classified using Kraken2 and our custom database. A) displays distribution of all classified reads B) displays how the classified reads are distributed.

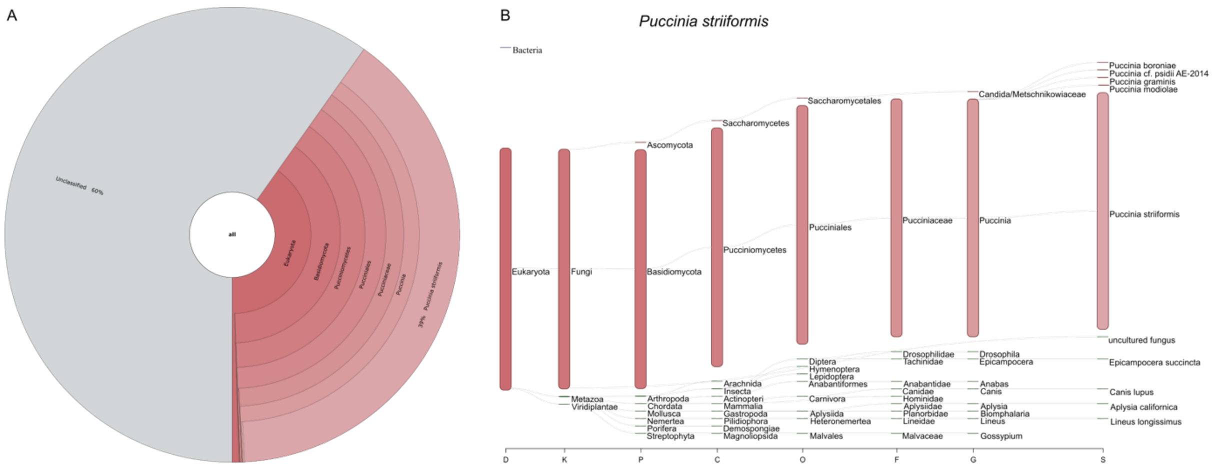


**Figure S7. Evaluation of our ability to classify DNA from *Puccinia striiformis*, Related to Table 1.**

Overview of how reads generated from the reference genome of *Puccinia striiformis* are classified using Kraken2 and our custom database. A) displays distribution of all classified reads B) displays how the classified reads are distributed.

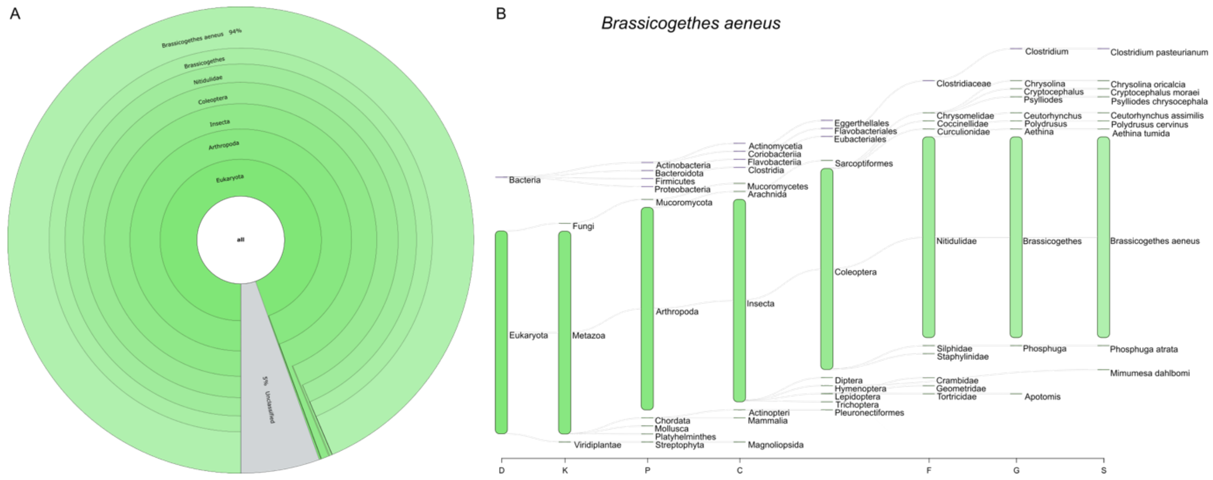


**Figure S8. Evaluation of our ability to classify DNA from *Brassicogethes aeneus*, Related to Table 1.**

Overview of how reads generated from the reference genome of *Brassicogethes aeneus* are classified using Kraken2 and our custom database. A) displays distribution of all classified reads B) displays how the classified reads are distributed.

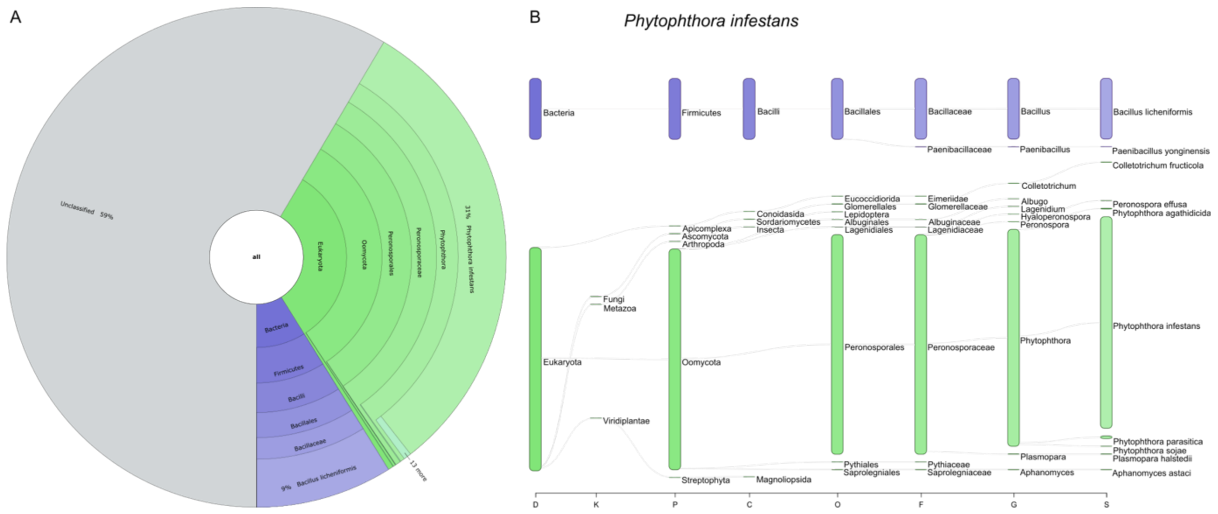


**Figure S9. Evaluation of our ability to classify DNA from *Phytophthora infestans*, Related to Table 1.**

Overview of how reads generated from the reference genome of *Phytophthora infestans* are classified using Kraken2 and our custom database. A) displays distribution of all classified reads B) displays how the classified reads are distributed.

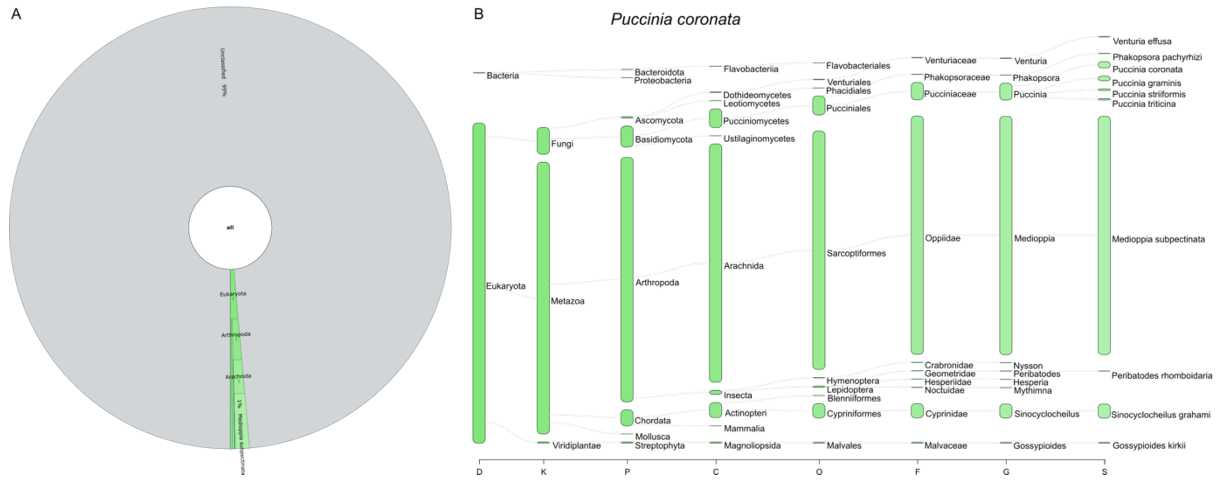


**Figure S10. Evaluation of our ability to classify DNA from *Puccinia coronata*, Related to Table 1.**

Overview of how reads generated from the reference genome of *Puccinia coronata* are classified using Kraken2 and our custom database. A) displays distribution of all classified reads B) displays how the classified reads are distributed.

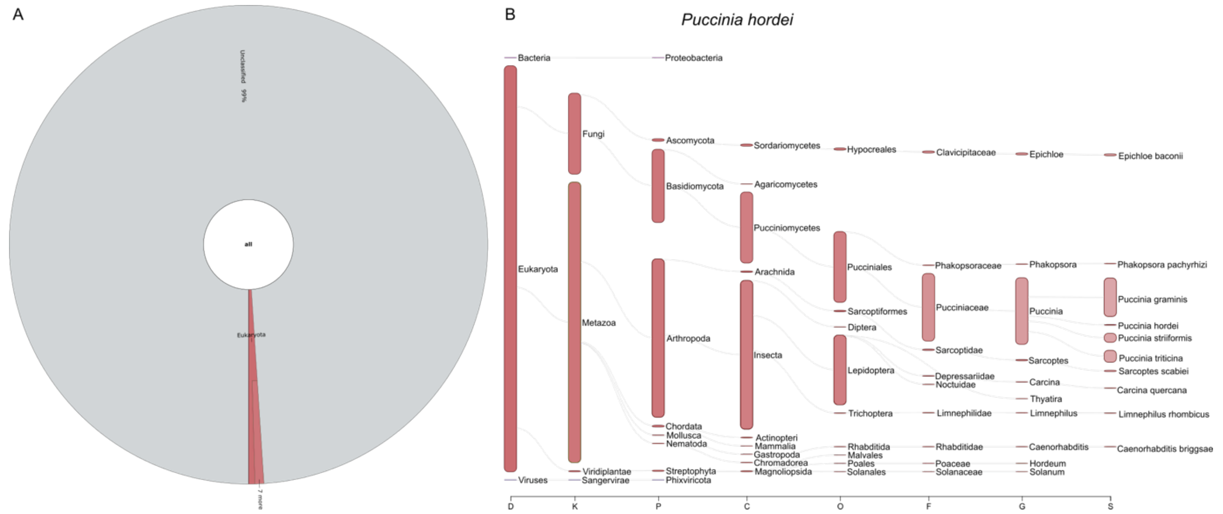


**Figure S11. Evaluation of our ability to classify DNA from *Puccinia hordei*, Related to Table 1.**

Overview of how reads generated from the reference genome of *Puccinia hordei* are classified using Kraken2 and our custom database. A) displays distribution of all classified reads B) displays how the classified reads are distributed.

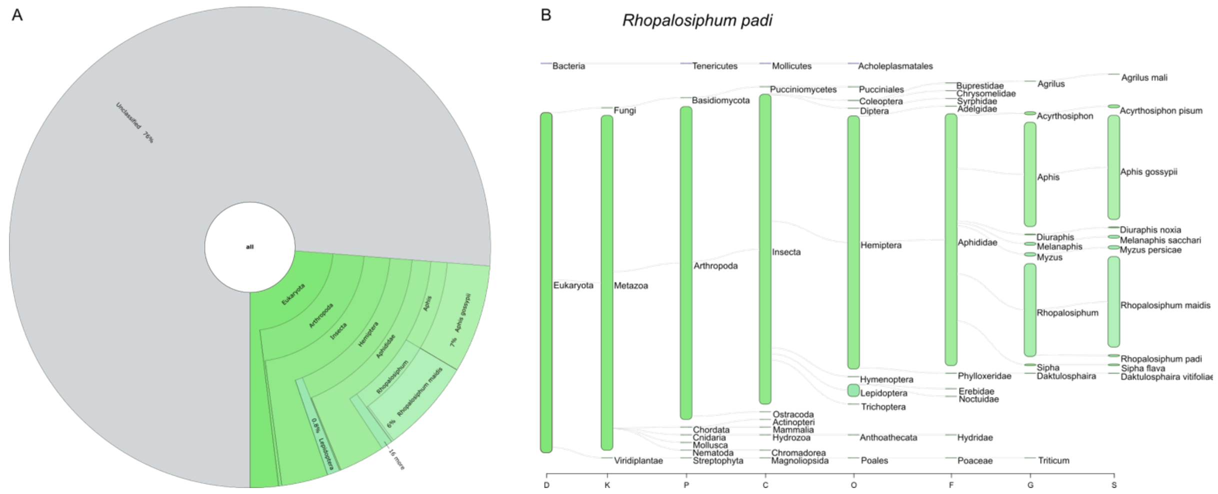


**Figure S12. Evaluation of our ability to classify DNA from *Rhopalosiphum padi*, Related to Table 1.**
Overview of how reads generated from the reference genome of *Rhopalosiphum padi* are classified using Kraken2 and our custom database. A) displays distribution of all classified reads B) displays how the classified reads are distributed.

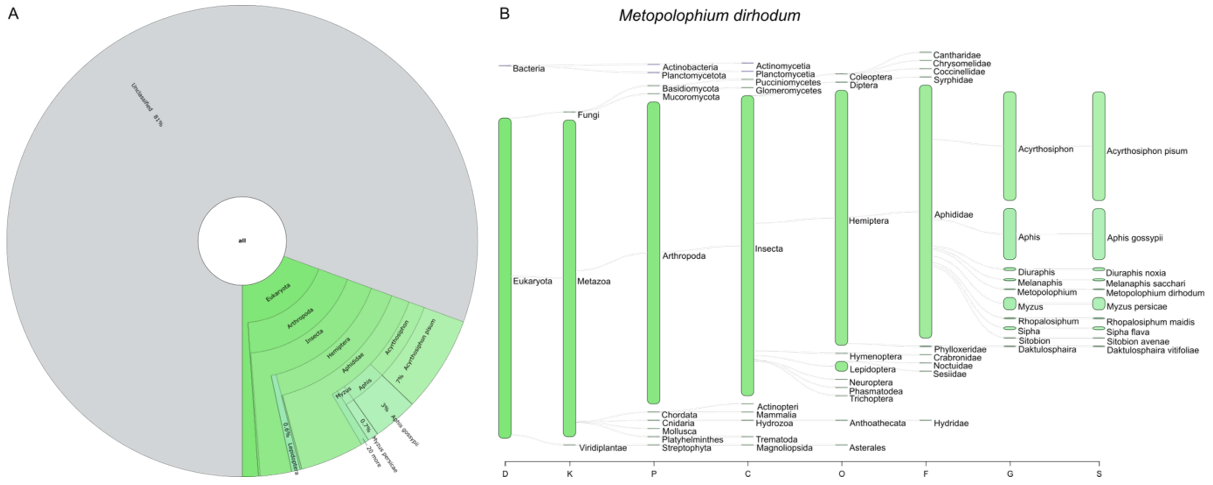


**Figure S13. Evaluation of our ability to classify DNA from *Metopolophium dirhodum*, Related to Table 1.**
Overview of how reads generated from the reference genome of *Metopolophium dirhodum* are classified using Kraken2 and our custom database. A) displays distribution of all classified reads B) displays how the classified reads are distributed.

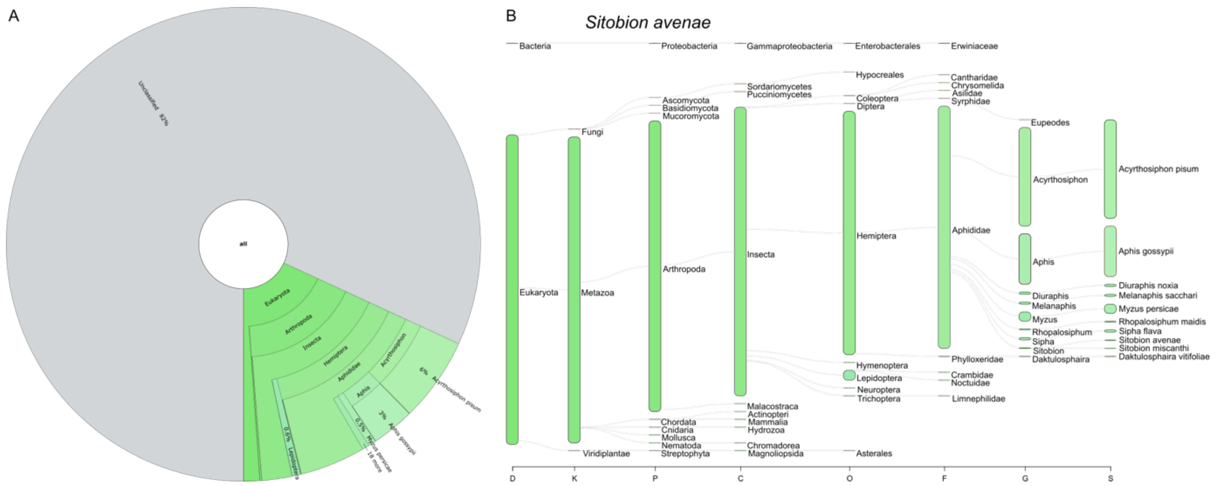


**Figure S14. Evaluation of our ability to classify DNA from *Sitobion avenae*, Related to Table 1.**

Overview of how reads generated from the reference genome of *Sitobion avenae* are classified using Kraken2 and our custom database. A) displays distribution of all classified reads B) displays how the classified reads are distributed.

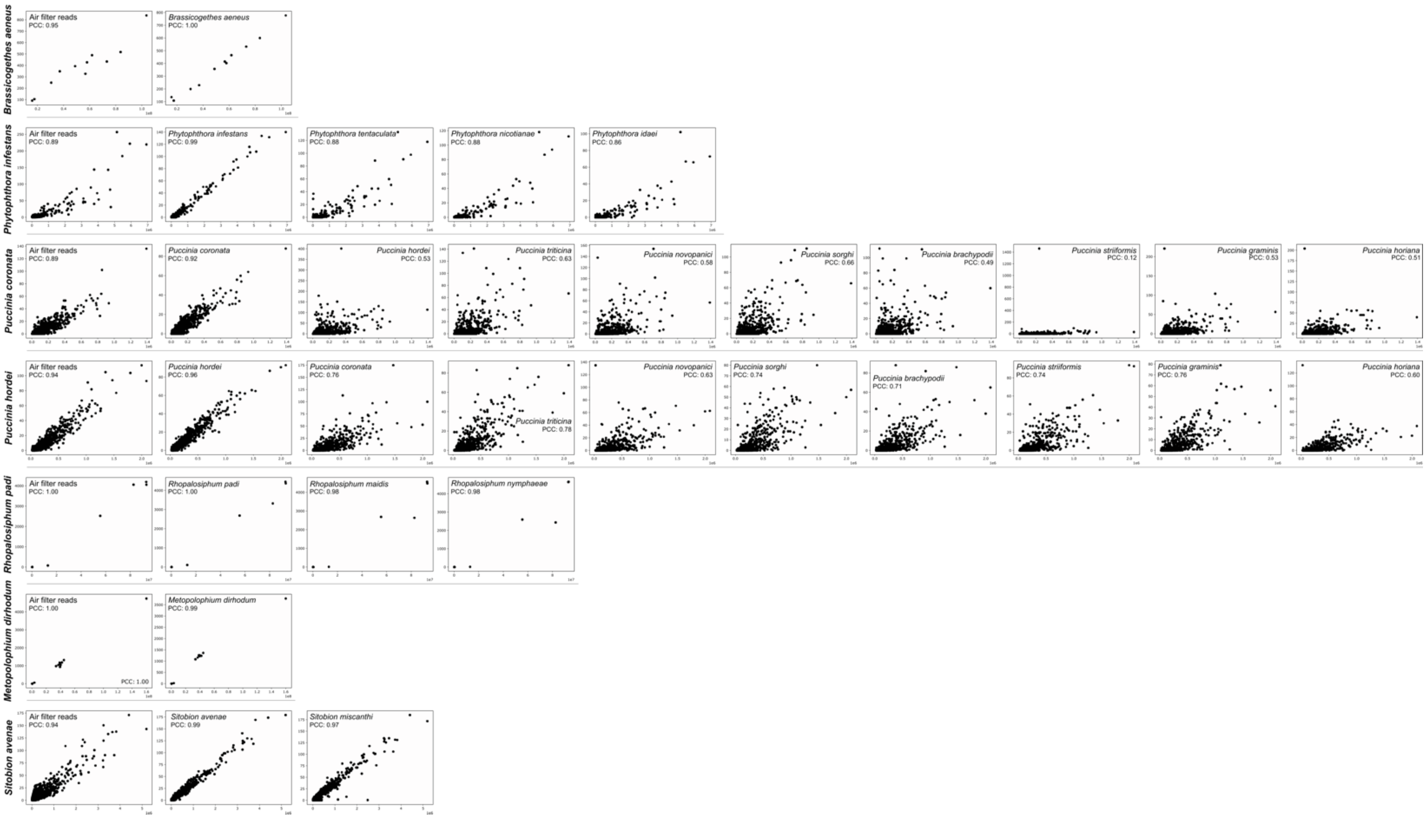


**Figure S15. Mapping to evaluate if DNA from lowly abundant species are present in the air filters, Related to Table 1.**

Correlation between **A.** mapped air filter reads and contig length **B.** mapped reads generated from the species reference genome **C.** mapped reads generated from other species from the same taxonomic genus. If correlations produced by the mapped air filter reads are stronger than any correlation produced by reads generated from other species, DNA from the lowly abundant species is deemed to be present in the air filters.

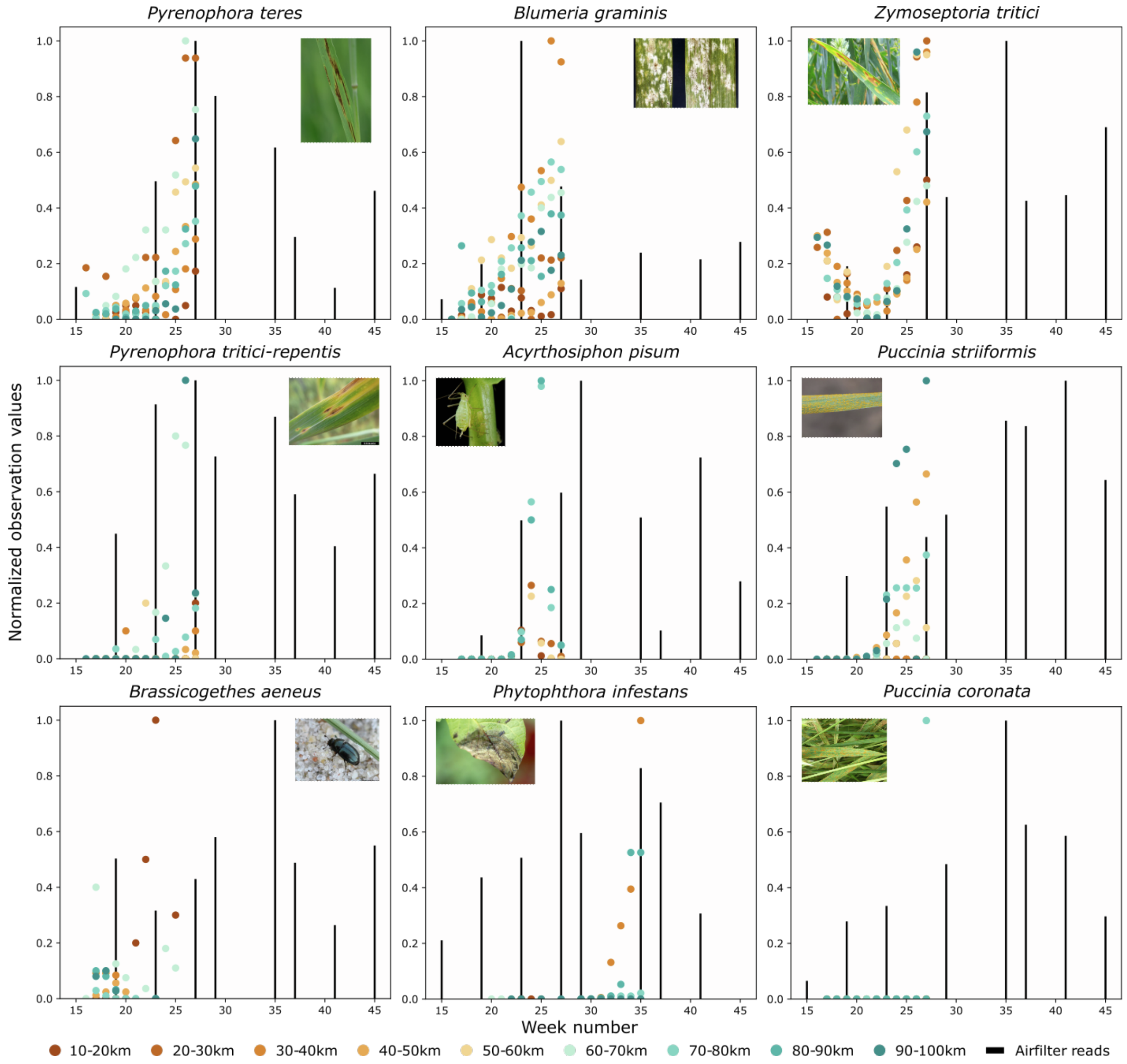


**Figure S16. Observed degree of damage correlate to signal strength, Related to Figure 4.** Average degree of damage caused by plant pathogens at different distance from the location where the air filters are collected. Data on observed degree of damage 2007 were obtained from the Swedish board of agriculture.
